# Environmental Triggers and Ocular Disease Networks: Analyzing the Impact of Air Pollutants and Meteorological Factors Using Fuzzy Cognitive Maps

**DOI:** 10.1021/envhealth.5c00522

**Published:** 2026-01-14

**Authors:** Li Zhang, Fabao Xu, Haixiang Jiang, Yaxin Miao, Yi Xiang, Lu Zhang, Qing Huang, Deying Yu, Meijia Wang, Xu Wang, Shiqiang Li, Boxuan Song, Zhiwen Li, Xueying Yang, Jing Wei, Jianqiao Li, Chengcheng Zhang, Kai Zhang, Yong Wang

**Affiliations:** † Department of Ophthalmology, 577528 The Central Hospital of Wuhan, Tongji Medical College, Huazhong University of Science and Technology, 26 Shengli Street, Jiang’an, Wuhan, Hubei 430014, China; ‡ 159369 Aier Eye Hospital of Wuhan University (Wuhan Aier Eye Hospital), No. 481, Zhongshan Road, Wuchang, Wuhan, Hubei 430060, China; § Wuhan Aier Eye Institute, No. 790, Minzhu Road, Wuchang, Wuhan, Hubei 430060, China; ∥ Department of Ophthalmology, 91623 Qilu Hospital, Shandong University, 107 West Wenhua Road, Lixia, Jinan, Shandong 250011, China; ⊥ Haixiang Eye Hospital, 44 Changle West Road, Xincheng, Xi’an, Shaanxi 710032, China; # Department of Ophthalmology, 69844 University Malaya Medical Centre, , Jalan Profesor Diraja Ungku Aziz, Lembah Pantai, Kuala Lumpur, Wilayah Persekutuan Kuala Lumpur 59100, Malaysia; ∇ School of Electronic Information and Artificial Intelligence, 74618 Shannxi University of Science & Technology, Xi’an Weiyang University Park, 1 Weiyang Road, Weiyang, Xi’an, Shaanxi 710021, China; ○ School of Software, 74600 Shanxi Agricultural University, 1 Mingxian South Road, Taigu, Jinzhong, Shanxi 030801, China; ◆ MEEKL-AERM, College of Environmental Sciences and Engineering, Institute of Tibetan Plateau, and Center for Environment and Health, 12465 Peking University, 5 Yiheyuan Road, Haidian, Beijing 100871, China; ¶ Medical Genetics Center, 477167 Maternal and Child Health Hospital of Hubei Province, 745 Wuluo Road, Hongshan, Wuhan, Hubei 430070, China; ⟁ 47905 Gyenno Science Co. Ltd. 18F, Tower B, Galaxy World, 1 Changfa Road, Nanshan, Shenzhen, Guangdong 518055, China

**Keywords:** environmental exposure, air pollution, climate change, ocular diseases, fuzzy cognitive maps (FCMs)

## Abstract

Growing evidence suggests that climate change and air pollution significantly contribute to the rising incidence of ocular morbidity; however, quantifying the complex interplay of these factors presents significant methodological challenges. This study aimed to investigate the impact of multiple air pollutants and meteorological conditions on common ocular diseases utilizing Fuzzy Cognitive Maps (FCMs) and to delineate the pathogenic relationships among them. We analyzed data from 28,981 hospitalizations in ophthalmology (2019–2024) at a tertiary hospital in Wuhan, China, coupled with daily environmental data for key air pollutants (PM_2.5_, PM_10_, SO_2_, NO_2_, CO, and O_3_) and meteorological parameters (temperature, humidity, atmospheric pressure, wind speed, precipitation, etc.). Eleven distinct FCM models were developed via differential evolution algorithms to quantify both environment–disease and disease–disease interactions. Our findings indicated that optic neuropathy (ON) exhibited the highest environmental sensitivity, showing a positive correlation with PM_10_. Besides, positive associations were identified between minimum temperature and glaucoma as well as between CO exposure or temperature and cataract risk. Diseases of the Ocular Surface and Appendages (DOSA) exhibited a robust correlation with precipitation, which was negatively associated with cataract and ON. Analysis of the disease interaction network identified thyroid-associated orbitopathy (TAO) as a central hub, subject to considerable input from other ocular conditions (ON → TAO: *n* = 59; LDD → TAO: *n* = 49; DOSA → TAO: *n* = 57; TAO → U: *n* = 42; TAO ↔ S: *w* = 0.45; G ↔ TAO: *w* = 0.37; OT ↔ TAO: *w* = 0.72). The most robust bidirectional association was observed between ON and Ocular Trauma (OT) (average weight = 0.93). Overall, this study highlights the effectiveness of FCM for modeling in environmental ophthalmology, revealing that PM_10_ exerts a critical influence on optic nerve health, while temperature and precipitation serve as vital modulators in glaucoma and cataract pathways. These findings should be interpreted as offering novel insights with implications focusing on future causal validation rather than establishing deterministic relationships. These insights provide quantitative evidence supporting the potential role of environmental factors in ocular diseases, thereby underscoring the need for targeted public health strategies and optimized medical resource allocation to mitigate the ocular health hazards posed by regionally prevalent and seasonal environmentally related eye disorders.

## Graphical abstract

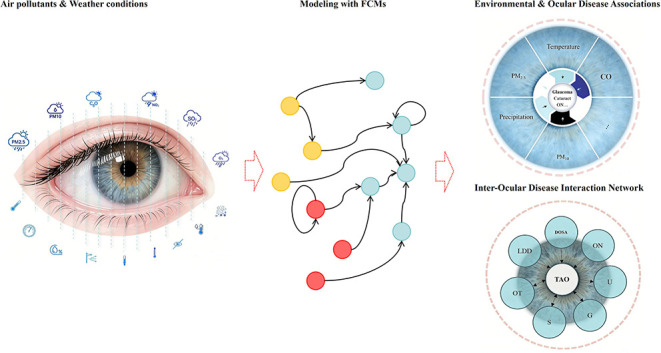

## Introduction

The rapid advancement of industrialization and urbanization has established air pollution and climate change as pivotal determinants of public health globally.^1^ Air pollution constitutes a major contributor to the global disease burden and has been recognized as the fifth leading risk factor for mortality worldwide.^2^ The profound health implications are underscored by the estimated 1.2 million deaths attributable to fossil fuel-derived air pollution in 2020 alone, while heat-related mortality demonstrated an alarming 68% increase between 2000 and 2004 and 2017–2021.^3^ The detrimental effects of climate change on biological functions are systemic, impacting functions ranging from the respiratory and cardiovascular systems to the nervous system,^4–6^ with emerging evidence indicating potentially significant consequences for visual health.^7^

There is an increasing consensus suggesting a significant association between environmental factors and ocular pathology.^8^ Substantial evidence indicates that air pollution and climate variations significantly influence the pathogenesis and progression of diverse ophthalmic conditions.^8,9^ Exposure to particulate matter (such as PM_2.5_ or PM_0.1_), environmental tobacco smoke, inadequate sunlight exposure, and elevated temperatures can disrupt immune homeostasis through Th17/Treg cell imbalance, macrophage polarization alterations, and neutrophil activation. These mechanisms subsequently trigger systemic inflammatory responses and oxidative stress, ultimately compromising ocular integrity through reduced retinal perfusion, enhanced tissue fibrosis, and sympathetic nervous system activation.^10^ Such pathways contribute to the pathogenesis of autoimmune ocular disorders,^11–15^ including uveitis, Graves’ ophthalmopathy, allergic conjunctivitis, glaucoma, and diabetic retinopathy. Furthermore, climate change-associated temperature increases and extreme weather events may amplify the risks of ocular allergies and infections,^1,16^ while stratospheric ozone depletion-mediated ultraviolet radiation exposure elevates susceptibility to cataracts,^17^ age-related macular degeneration,^18^ and pterygium.^19^

Conventional epidemiological approaches have predominantly examined isolated environmental variables in relation to specific ocular conditions, thereby limiting a comprehensive understanding of multifactorial interactions. In this context, Fuzzy Cognitive Maps (FCMs) serve as a transformative framework for modeling complex system dynamics.^20^ Recent advances in environmental modeling, such as wavelet artificial neural networks for PM2.5 prediction and deep learning approaches for climate forecasting, have enhanced our ability to quantify complex environmental exposures, providing a robust foundation for integrating high-resolution data into health studies.^21,22^ This computational methodology integrates multivariate interactions through temporal modeling, incorporating self-feedback mechanisms, and constructing visualized networks of concept nodes and weighted directional edges that explicitly represent component interrelationships. Accordingly, FCMs facilitate both qualitative and quantitative analysis of disease interactions, thereby advancing pathogenetic insight, facilitating incidence forecasting, and informing the development of novel environmentally oriented prevention strategies.

Despite growing recognition of environmental-ocular health connections, the precise mechanisms and magnitude of the effects remain inadequately characterized. To our knowledge, this is the first study to adopt an FCM approach to quantitatively analyze the effects of meteorological conditions and air pollution on ophthalmic disease patterns among hospitalized patients, while concurrently exploring potential interdisease relationships. Our findings aim to establish an evidence-based foundation for public health policy formulation and clinical pathway optimization in environmental ophthalmology.

## Material and Methods

### Data Collection and Characteristics

The data utilized in this study were extracted from the in-patient records of individuals treated at The Central Hospital of Wuhan from January 1, 2019 to June 30, 2024. A total of 28981 patients with one of ten common ocular diseases were included. The disease categories were as follows: Vitreoretinal Diseases (VRD), Thyroid-Associated Orbitopathy (TAO), Cataract (C), Lacrimal Duct Diseases (LDDs), Uveitis (U), Glaucoma (G), Strabismus (S), Diseases of Ocular Surface and Appendages (DOSA), Optic Neuropathy (ON), and Ocular Trauma (OT). To ensure data quality, diagnostic reliability was supported by the tertiary hospital setting, where all initial diagnoses were established by attending ophthalmologists and subsequently validated during ward rounds by associate or chief physicians. Furthermore, although inherent coding variations may occur at the subcategory level, all cases assigned to the 10 major ophthalmic disease categories underwent systematic verification and multitier review during the classification process. This rigorous protocol effectively mitigated potential misclassification biases associated with large-scale electronic health records. Besides, meteorological and air quality data for the corresponding period in Wuhan were sourced from online platforms (https://rp5.ru/ and https://www.aqistudy.cn/), with a statistical overview of the data set provided in Table S1. The environmental factors comprised six air pollutants (PM_2.5_, PM_10_, SO_2_, CO, NO_2_, and O_3_) as well as nine meteorological factors (temperature, atmospheric pressure, humidity, wind speed, minimum temperature, maximum temperature, visibility, dew point, and precipitation). The measurement requirements for meteorological parameters are as follows: air temperature and dew point were measured at 2 m above ground level; minimum and maximum temperatures represent the lowest and highest values, respectively, recorded over the preceding 12-h period; and wind speed denotes the maximum gust speed measured 10–12 m above ground level within a 10 min interval. Furthermore, given that more than ten measurements were recorded daily, the daily value was calculated as the 24-h average. The O_3_ concentration was reported as the maximum 8-h moving average. Clinical data were collected from the Department of Ophthalmology at the Central Hospital of Wuhan. Weather and air quality data were acquired from two validated online sources. The study received approval from the Ethics Committee of the Central Hospital of Wuhan (Ethics Number: WHZXKYL2025-129) and adhered to the principles outlined in the Declaration of Helsinki. Prior to modeling, all factors underwent min–max normalization. The initial adjacency matrix for each Fuzzy Cognitive Map was generated randomly and then optimized using a differential evolution algorithm to fit the time series data. Each model was independently constructed ten times, and the one with the optimal fitness was selected for final analysis, ensuring robustness and minimizing reliance on initial conditions. Throughout the entire computational procedure, no manual intervention was involved. The first three-quarters of the data set were designated as the training set, and the last quarter was designated as the test set.

A total of 11 FCM models were developed in this study (Figure 1). Models 1 through 10 were designated for the singular model analysis, wherein each model focused on a specific disease while incorporating all environmental factors. Model 11 was reserved for the Overall Model Study, encompassing all disease types and environmental factors. Each model was iterated ten times, and the model exhibiting the best fitness was selected as the final representative model.

**Figure 1:**
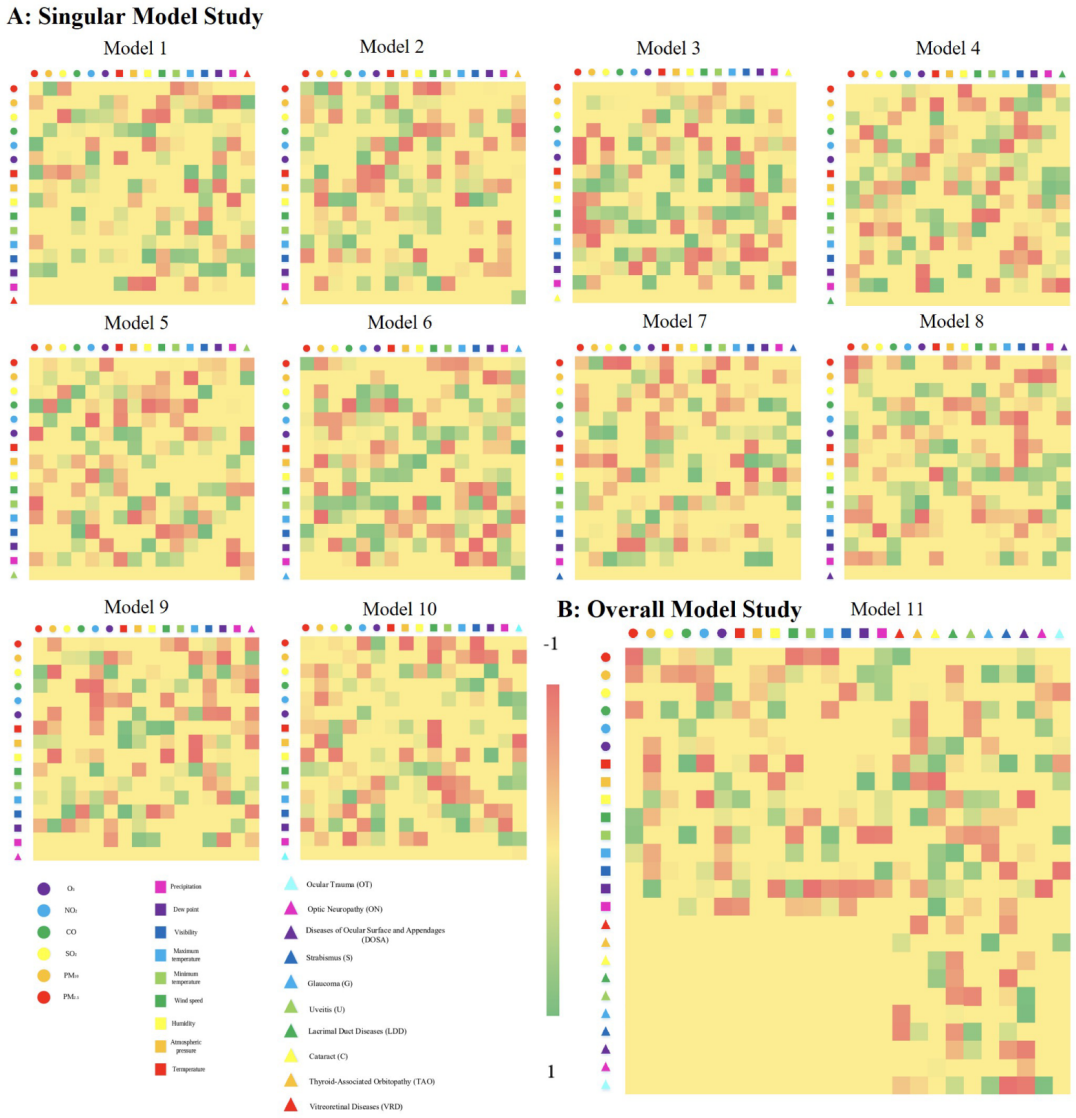
The final graphs (FCMs) for ten categories of ocular diseases in both the singular (A) and the overall model study (B). Model 1 for Vitreoretinal Diseases (VRDs); model 2 for Thyroid-Associated Orbitopathy (TAO); model 3 for Cataract (C); model 4 for Lacrimal Duct Diseases (LDDs); model 5 for Uveitis (U); model 6 for Glaucoma (G); model 7 for Strabismus (S); model 8 for Disease of Ocular Surface and Appendages (DOSA); model 9 for Optic Neuropathy (ON); model 10 for Ocular Trauma (OT); model 11 for all diseases.

### Fuzzy Cognitive Maps

FCMs describe a set of concepts (nodes) and the interrelationship between them,^23,24^ forming a directed cyclic graph *G* = (*C*,*E*,*U*,*f*), where *C* = (*c*_1_,*c*_2_, ...,*c_n_*) denotes the set of concepts that constitute the nodes of the directed cyclic graph and *n* is the number of variables in the model. *E* denotes the set of weights *e_ij_* on the directed edge from the concept node *c_i_* to the concept node *c_j_* (1 ≤ *i*,*j* ≤ *n*), namely, *E* is an adjacent matrix. In this study, all nodes represent the climatic and air quality indicators and the number of cases for each ocular disease summarized daily. To evaluate potential multicollinearity among variables and visualize their complex relationships within the model, we summarized and analyzed all simple paths between concepts (see Figure S1). This analysis helped elucidate the interaction networks among environmental indicators, among ophthalmic diseases, and between diseases and environmental factors, providing a foundation for the interpretation of subsequent results. The FCM modeling process is formulated as an optimization task to infer the adjacent matrix *E* to fit the observational values along the time axis (the time series prediction involves multiple variables). *U, U*(*t*) = (*u*_1_(*t*_0_), *u*_2_(*t*_0_),..., *u*_c_(*t*_0_)), is the observational values of all concepts (nodes) at the initial time *t*_0_. The function *f* denotes the transition function that calculates the predicted value of a conceptual node *i* at time *t + 1* from the observed values of all conceptual nodes at time *t*. The prediction for all nodes (concepts) at time *t + 1* is shown as eq 1, where $f\left(x\right)=\frac{1}{1+e^{‐\lambda x}},\lambda =5$ . In our previous study, we have verified that *λ* has negligible impact on the experimental results.^24^

1 $$u_{i}\left(t+1\right)=f\left(\overset{c}{\underset{j=1}{\sum }}e_{ji}u_{j}\left(t\right)\right)$$

Similar to the conventional time series prediction, the order of FCM defines the latency of all variables (concepts); namely, first-order FCM indicates that the values of all nodes at time stamp *t* depend on the values of all nodes at time stamp *t – 1*. A second-order FCM indicates that the values of all nodes at time stamp *t* are related to the values of all nodes at time stamps *t – 1* and *t – 2,* and so on. In this study, we constructed first-, second-, and third-order FCMs to capture the complicated relationship between all variables and selected the model with optimal fitting performance (the best fitness value during the differential evolution optimization process) as the final FCM. This design inherently incorporates lag-effect into the modeling framework.

### Differential Evolution Algorithm

We adopted the Differential Evolution (DE)^25^ algorithm, which has been proven effective in numerical optimization problems, to optimize the FCMs. DE applies a virtual population formed of some individuals that simulate evolutionary processes to iteratively refine the solution toward the optimal. Each individual is represented as a vector corresponding to the values in *E* of the FCMs, specifically the adjacency matrix of the FCM (graph). In each iteration, individuals (analogous to chromosomes in DE) undergo crossover and mutation operations to generate mutated individuals. Then, the original and mutated individuals are compared via greedy selection to form the new population, where the individual with the better value from the objective function is retained. The objective function aims to optimize the adjacency matrix *E* to fit the historical observational data. To account for the heterogeneity of each monitoring site across different years, the objective function is defined to minimize the discrepancy between predicted and observed values, as shown in eq 2, where *u_i_* (*t*) and *u_i_* (*t*)′ denote the predicted value and observed value of the *i*th node at time *t*. *T* is the total number of time stamps. The crossover and mutation operations are shown in eqs 3 and 4, where *v*_i_ is the *i*th individual and *v*_1_, *v*_2_, and F ∈ [0,1] represent two randomly selected individuals and the cross factor (an algorithm parameter), respectively. *v*_i.j_ is the *j*th element in the *i*th individual, *rand*(), *v*_r,j_ and CR are a random scalar in [0,1], the *j*th element in a randomly selected individual *v*_r_ and the mutation rate (an algorithm parameter), respectively. The mutation operation defined in eq 4 ensures that at least one element in the original individual undergoes a mutation. Specifically, if all elements in *v*_i_ are not mutated, the selected *j*th element will be mutated. *n* is the number of indicators in the present study, which is consistent with *n* in the FCM section. The DE algorithm terminates its iterative computation when the maximum epoch is reached or upon achieving convergence. All algorithm parameters are listed in Table S2. In this research, an individual in the DE algorithm represents the adjacency matrix *E*, and the predictive values after time stamp *t*_0_, *u_i_* (*t* > *t*_0_), are computed via eq 1 for optimization. Given that the objective of this study is to investigate the impact of multiple air pollutants and meteorological factors on ten categories of ocular diseases utilizing FCMs and to delineate the interpathology relationships using FCMs, the feedback effect of disease incidence on environmental factors was excluded from the DE optimization process.

$$L=\overset{n}{\underset{i=1}{\sum }}\overset{T}{\underset{t=1}{\sum }}{\left(u_{i}\left(t\right)-u_{i}{\left(t\right)}^{\prime }\right)}^{2}$$ 2

3 $$v_{i}=v_{i}+F\left(v_{1}-v_{2}\right)$$

4 $$v_{i.j}=v_{r,j},if\;rand\left(\right)\leq CR\;or\;j=rand\left(1,n^{2}\right)$$

All code is implemented with MATLAB R2016 and run on a server with 64 CPUs (Intel­(R) Xeon­(R) Gold 5218 CPU at 2.30 GHz) and 256 GB of memory. Because FCMs and DE cost much time to optimize the results, all parameters in this research are selected based on our previous research and experience.^23,24,26^

### Singular or Overall Model Study

Within the framework of the Singular Model Study, the influence of environmental factors on distinct ocular diseases was assessed using ten individualized models (Models 1 to 10), with each model representing a particular disease. In contrast, the Overall Model Study employed an integrated approach (Model 11) to comprehensively evaluate environmental effects across diverse ocular disorders and simultaneously investigate potential underlying relationships among these conditions.

## Results

First-, second-, and third-order FCMs were constructed using the DE algorithm, and the model demonstrating optimal performance (with the lowest fitness value) was selected as the final model. A summary of the fitness values is provided in Table S3. To further evaluate the model accuracy, we also provided the Mean Absolute Error (MAE) for all 11 models in Table S4.

### Demographic Analysis and Screening

A total of 28,981 patients were included in this study, with 3,674 enrolled in 2024 (1,637 males and 2,037 females), 7,258 in 2023 (3,176 males and 4,082 females), 4,538 in 2022 (2,041 males and 2,497 females), 5,002 in 2021 (2,336 males and 2,666 females), 2,523 in 2020 (1,193 males and 1,330 females), and 5,986 in 2019 (2,753 males and 3,233 females). The mean age of the study population was 65.89 ± 12.98 years. The diseases studied were categorized into ten primary groups: 1. vitreoretinal diseases (*n* = 7836 cases); 2. thyroid-associated orbitopathy (*n* = 58 cases); 3. cataract (*n* = 16351 cases); 4. lacrimal duct diseases (*n* = 363 cases); 5. uveitis (*n* = 248 cases); 6. glaucoma (*n* = 921 cases); 7. strabismus (*n* = 93 cases); 8. disease of the ocular surface and appendages (*n* = 2561 cases); 9. optic neuropathy (*n* = 54 cases); 10. ocular trauma (*n* = 496 cases). Table S5 summarizes the demographic characteristics, including the average age and sex distributions of all patients. Table S6 presents the Variance Inflation Factor (VIF) values^27^ used to evaluate multicollinearity among the data set variables. Notably, a strong correlation was observed between PM_2.5_ and PM_10_, as anticipated. In addition, meteorological variables—including temperature, humidity, minimum and maximum temperatures, and dew point—exhibited substantial collinearity. Despite the presence of multicollinearity in the data set, fuzzy cognitive mapping (FCM) remains capable of identifying underlying interrelationships.

Singular Model Study: Utilizing models 1–10, we assessed the effects of environmental factors on various eye diseases. The distribution of weights for the simple paths within these models 1–10 is shown in Figure S2A. Analysis using the Number of Simple Paths as a metric (which calculates the average environmental impact on each disease type and ranks them in descending order) identified glaucoma as the most susceptible condition (*n* = 326), followed by ON (*n* = 249), LDD (*n* = 202), OT (*n* = 187), DOSA (*n* = 174), C (*n* = 159), S (*n* = 145), VRD (*n* = 139), U (*n* = 132), and TAO (*n* = 118) (Figure 2A). Furthermore, ranking by the Absolute Mean of Simple Path Weights identified ON (mean weight = 0.00354) as the most markedly influenced condition, followed by VRD (0.002199), C (0.00319), TAO (0.00186), U (0.00177), S (0.001123), LDD (0.00071), DOSA (0.000221), G (0.000213), and OT (0.000131) (Table 1).

**Table 1:** Weights of Simple Paths for Ten Ocular Diseases in the Singular Model Study^a^

Parameters	Weights									
**Models**	Model 1	Model 2	Model 3	Model 4	Model 5	Model 6	Model 7	Model 8	Model 9	Model 10
**Ocular diseases**	VRD	TAO	C	LDD	U	G	S	GOSA	ON	OT
**PM**_**2.5**_	–0.005805	–0.01023	0.001204	–0.01335	0.00432	0.003644	–0.00333	–0.00667	–0.01805	–0.01083
**PM**_**10**_	–0.00284	–0.01463	–0.00352	0.000104	0.002625	0.00657	0.005539	0.019615	0.004936	0.01964
**SO**_**2**_	–0.00921	–0.00381	0.008583	0.00354	–0.00531	–0.00535	–0.0062	–0.003	–0.01522	–0.0264
**CO**	0.007763	–0.00821	0.001447	0.006503	–0.00359	–0.00919	–0.01067	–0.00277	–0.00206	–0.01255
**NO**_**2**_	0.001842	–0.00803	–0.00426	–0.00057	–0.00577	0.010048	0.014304	–0.00787	–0.00075	0.01145
**O**_**3**_	–0.00369	0.002252	–0.02244	0.000579	0.002812	–0.00075	0.005943	–0.00678	–0.01385	0.051652
**Temperature**	0.00105	–0.01183	0.002767	0.000887	0.010841	–0.00562	0.0065	0.008473	0.009151	–0.00737
**Atmospheric pressure**	0.001273	0.002698	–0.00661	–0.00712	–0.00153	–0.00956	0.006052	0.00786	0.005577	–0.00892
**Humidity**	0.000996	0.009438	0.002567	0.000702	–0.01517	–0.00357	–0.00888	0.008226	–0.00939	–0.00281
**Wind speed**	0.006129	0.005645	–0.01165	–0.00967	–0.02159	0.010558	–0.00775	0.004211	0.01182	–0.0018
**Minimum temperature**	0.013096	–0.00354	–0.00388	–0.00164	–0.0037	–0.00014	0.022292	–0.01359	0.000601	0.000687
**Maximum temperature**	–0.01214	0.005839	–0.00172	–0.00485	0.011891	–0.00766	–0.00334	0.005314	–0.01623	–0.0162
**Visibility**	5.67 × 10^–05^	–0.003	0.011003	0.018036	–0.00536	–0.00246	0.005578	–0.00403	–0.00346	0.019572
**Dew point**	0.024834	0.001695	–0.00943	–0.00273	0.010258	0.006058	–0.01826	–0.00247	0.020644	–0.0104
**Precipitation**	0.009625	0.007763	–0.01198	–0.00114	–0.00727	0.010622	0.009062	–0.0032	–0.02683	–0.00375
**Absolute mean**	0.0032394	0.00186	0.00319	0.00071	0.00177	0.000213	0.001123	0.000221	0.00354	0.000131

a Models 1–10 and corresponding ocular diseases: model 1 for Vitreoretinal Diseases (VRDs); model 2 for Thyroid-Associated Orbitopathy (TAO); model 3 for Cataract (C); model 4 for Lacrimal Duct Diseases (LDDs); model 5 for Uveitis (U); model 6 for Glaucoma (G); model 7 for Strabismus (S); model 8 for Disease of Ocular Surface and Appendages (DOSA); model 9 for Optic Neuropathy (ON); model 10 for Ocular Trauma (OT).

**Figure 2:**
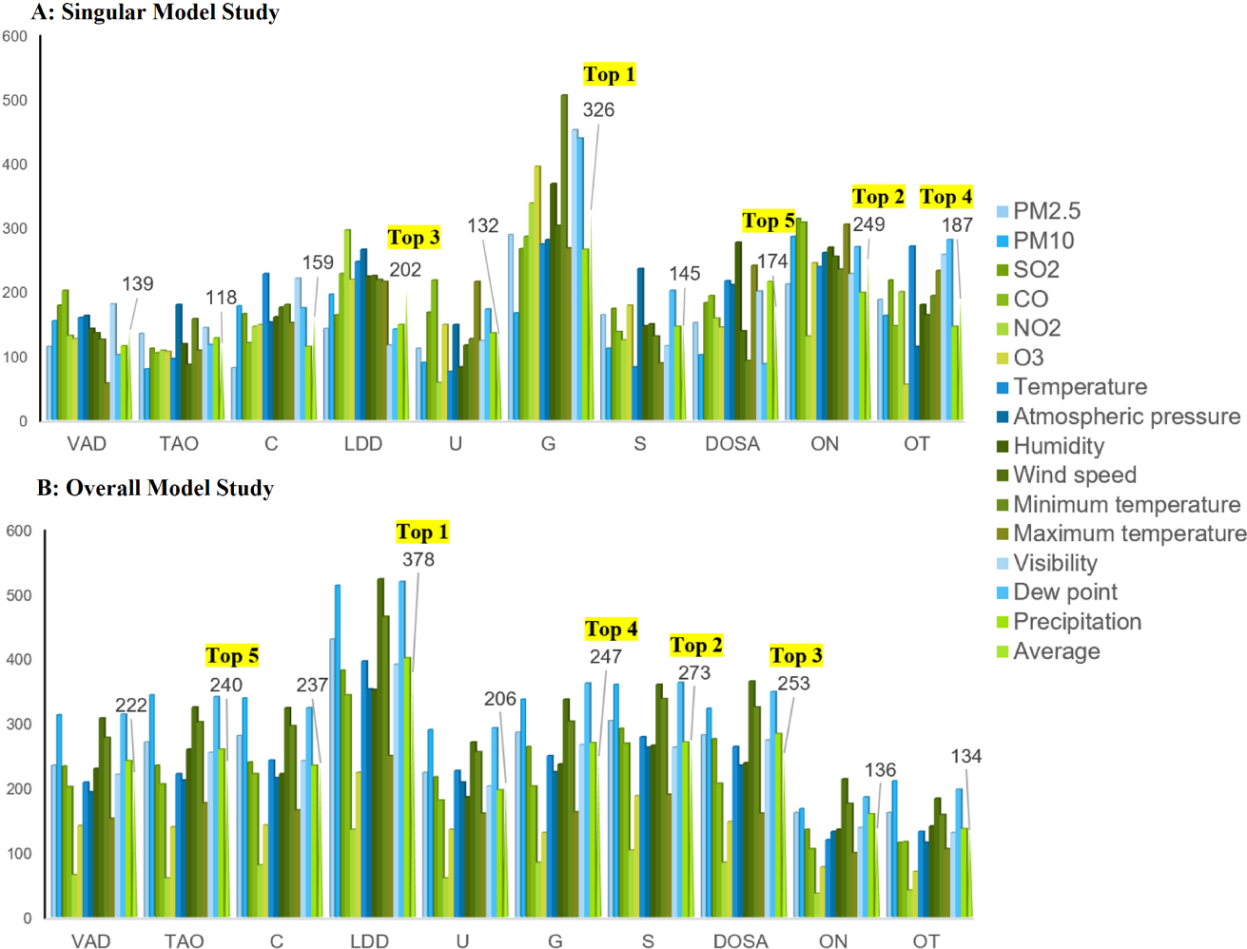
Mean number of simple paths for ocular diseases in the Singular and Overall Model studies. A (Singular Model): Top five diseases ranked by the mean number of simple paths in descending order: G (1st), ON (2nd), LDD (3rd), OT (4th), and DOSA (5th). B (Overall Model): Top five diseases ranked by the mean number of simple paths (descending order): LDD (1st), S (2nd), DOSA (3rd), G (4th), and TAO (5th). VRDs = Vitreoretinal Diseases; TAO = Thyroid-Associated Orbitopathy; C = Cataract; LDDs = Lacrimal Duct Diseases; U = Uveitis; G = Glaucoma; S = Strabismus; DOSA = Diseases of Ocular Surface and Appendages; ON = Optic Neuropathy; OT = Ocular Trauma.

Overall Model Study: The impact of environmental factors on ocular diseases was comprehensively analyzed using Model 11. Figure S2B displays the box plots of the simple path weights for this model. Evaluation using the Number of Simple Paths metric (which averages the influence of environmental factors on each disease type) identified LDD (*n* = 378) as the most susceptible condition, followed by S (*n* = 273), DOSA (*n* = 253), G (*n* = 247), TAO (*n* = 240), C (*n* = 237), VRD (*n* = 222), U (*n* = 206), ON (*n* = 136), and OT (*n*= 134) (Figure 2B). Conversely, ranking using the Absolute Values of Simple Path Weights identified ON (mean weight = 0.00589) as the most markedly influenced condition, followed by G (0.00376), S (0.003712), VRD (0.00208), C (0.001946), U (0.001792), OT (0.00139), TAO (0.00089), LDD (0.00083), and DOSA (0.000488) (Table 2).

**Table 2:** Weights of Simple Paths for Ten Ocular Diseases in the Overall Model Study^a^

Parameters	Weights									
**Models**	Model 11									
**Ocular diseases**	VRD	TAO	LAD	LDD	U	G	S	GOSA	ON	OT
**PM**_**2.5**_	0.010621	–0.0015	–0.01616	–0.00394	–0.00202	–0.00537	0.001522	0.01161	–0.00936	0.003694
**PM**_**10**_	–0.00074	0.005108	–0.00758	0.000921	0.009022	0.014237	–0.00781	0.0075	0.011872	0.012653
**SO**_**2**_	–0.00392	0.005742	–0.00178	0.000716	0.005047	0.001271	0.001852	0.004322	–0.00549	0.011138
**CO**	–0.01338	0.010233	0.004159	–0.00398	0.01783	–0.00475	0.007262	–0.01057	–0.0173	–0.00114
**NO**_**2**_	–0.003	–0.01382	0.013053	–0.00469	–0.01044	–0.0193	0.024574	–0.00703	–0.01722	–0.01519
**O**_**3**_	0.005918	–0.02073	0.013948	–0.00027	–0.00692	–0.01594	–0.00045	–0.02007	–0.00474	–0.02143
**Temperature**	–0.02631	0.011442	0.016658	–0.00627	0.005328	–0.00244	0.020151	0.000888	–0.00434	0.011881
**Atmospheric pressure**	0.017395	–0.02619	–0.0087	0.009219	–0.00598	–0.01707	–0.00613	–0.00674	–0.00991	–0.01588
**Humidity**	–0.0081	0.019742	–0.00375	–0.01291	0.013094	0.014596	0.012075	0.008854	0.007661	0.011563
**Wind speed**	–0.00319	0.001746	–5.7 × 10^–06^	–0.00016	0.00326	0.00164	–0.00031	0.011442	–0.01111	0.001895
**Minimum temperature**	–0.00282	–0.00058	0.013913	–5 × 10^–05^	–0.01013	–0.00992	0.012061	–0.00282	–0.00383	–0.01682
**Maximum temperature**	–0.00249	–0.01567	0.023572	0.005362	–0.0001	–0.00721	–0.00715	–0.01161	–0.00411	–0.01258
**Visibility**	–0.00086	–0.00742	6.6 × 10^–06^	–0.00664	0.002204	–0.00158	0.001976	0.002637	–0.00786	–0.01216
**Dew point**	0.000487	0.015156	–0.01378	2.26 × 10^–05^	0.007682	0.001855	–0.00138	0.013852	–0.00499	0.011635
**Precipitation**	–0.00087	0.003423	–0.00436	0.010205	–0.00101	–0.00636	–0.00257	0.005043	–0.00768	0.009948
**Absolute mean**	0.00208	0.00089	0.001946	0.00083	0.001792	0.00376	0.003712	0.000488	0.00589	0.00139

a Model 11 for all kinds of ocular diseases. VRDs = Vitreoretinal Diseases; TAO = Thyroid-Associated Orbitopathy; C = Cataract; LDD = Lacrimal Duct Diseases; U = Uveitis; G = Glaucoma; S = Strabismus; DOSA = Diseases of Ocular Surface and Appendages; ON = Optic Neuropathy; OT = Ocular Trauma.

Comprehensive Screening: By synthesizing the results from both the Singular and Overall model studies, results with an elevated number of simple paths and absolute weights were prioritized (Top 5). The flowchart illustrating data screening is presented in Figure 3.

**Figure 3:**
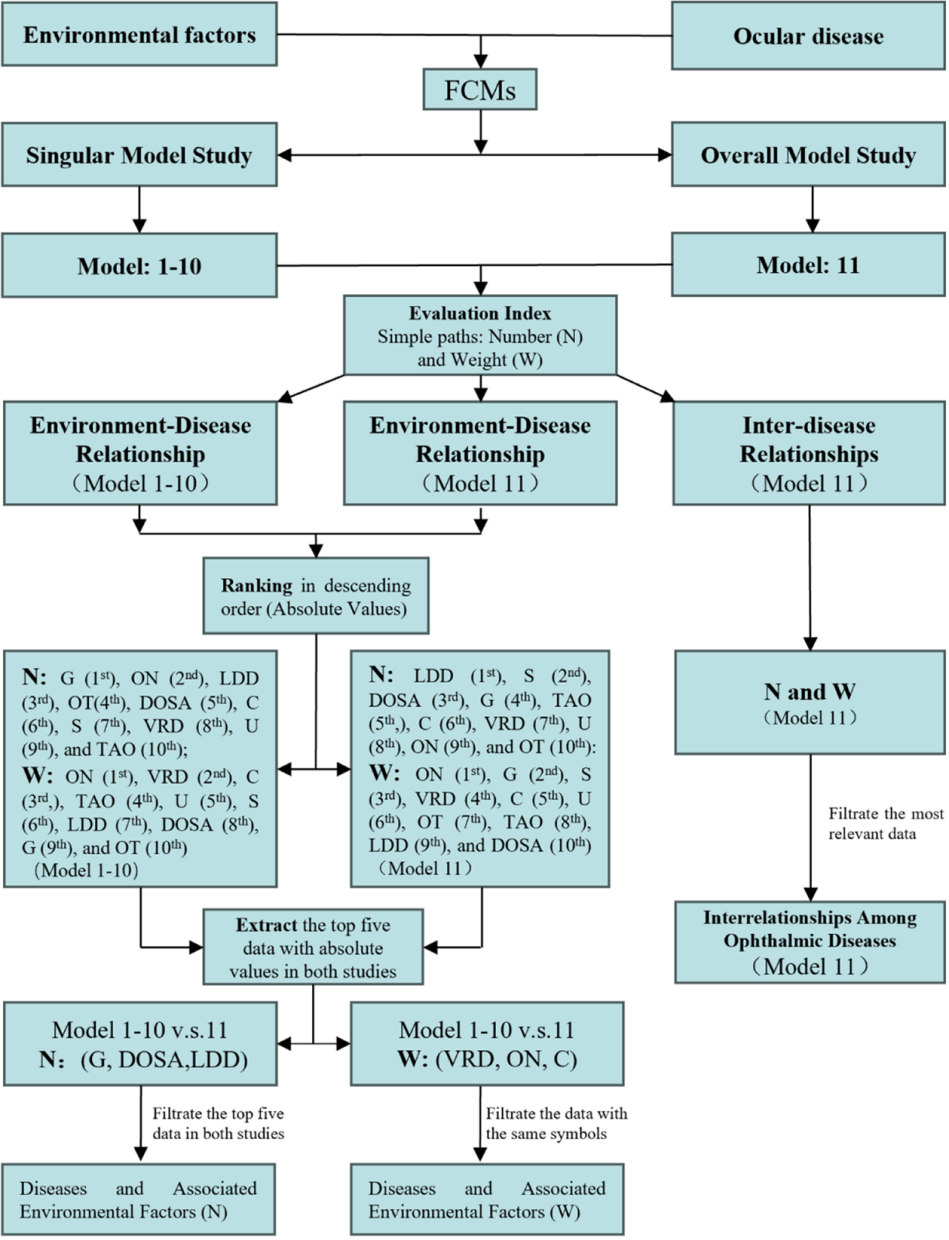
Flowchart of FCMs-based screening for environment-ophthalmic disease relationships. VRDs = Vitreoretinal Diseases; TAO = Thyroid-Associated Orbitopathy; C = Cataract; LDDs = Lacrimal Duct Diseases; U = Uveitis; G = Glaucoma; S = Strabismus; DOSA = Diseases of Ocular Surface and Appendages; ON = Optic Neuropathy; OT = Ocular Trauma; *N* = number; *W* = weight.

Explanation of Model Discrepancies and the Comprehensive Screening Rationale: The observed discrepancies in disease susceptibility rankings between the Singular and Overall Model analyses stem from their distinct analytical frameworks and objectives. The Singular Model approach (Models 1–10) examines each disease in isolation, quantifying the direct environmental influences specific to each condition. In contrast, the Overall Model (Model 11) captures the complex web of interactions among all diseases and environmental factors simultaneously, representing a systems-level perspective in which diseases influence each other through shared pathways.

Our comprehensive screening approach, which prioritizes associations based on both path count and absolute weight, is justified by the complementary nature of these metrics. Path count reflects the breadth of environmental connections (number of significant pathways), indicating how extensively a disease is influenced by various environmental factors. Absolute weight measures the strength of these influences (magnitude of the effect). By integrating both criteria, we identify diseases that are both widely connected to environmental factors and strongly affected by them, ensuring more robust and clinically relevant potential prioritization for future research and intervention strategies.

### Environmental Factors and Ocular Diseases

An analysis of the number of simple paths revealed several significant associations. Glaucoma was found to be associated with a minimum temperature and dew point. DOSA exhibited a correlation with precipitation. LDD displayed a relationship with wind speed. A weighted analysis of simple paths indicated significant positive correlations between VRD and both atmospheric pressure and the dew point. ON showed notable associations with environmental pollution and fluctuations in weather, being positively correlated with PM_10_ but negatively correlated with PM_2.5_, SO_2_, CO, NO_2_ and O_3_. Besides, ON demonstrated negative correlations with dew point, precipitation, maximum temperature, and visibility. Cataract was positively correlated with CO, visibility, and temperature, while exhibiting negative correlations with dew point and precipitation (Figure 4A)

**Figure 4:**
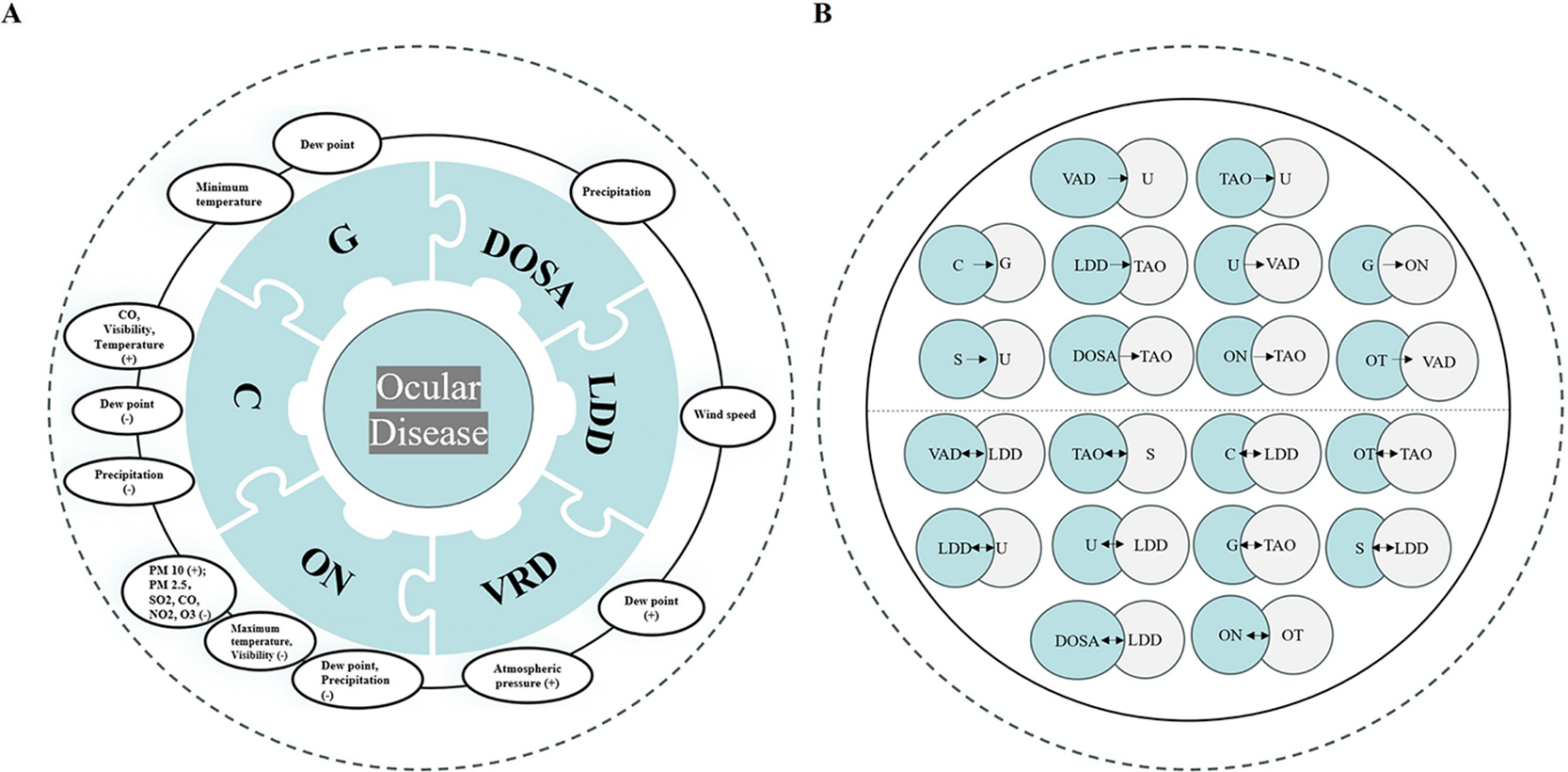
Environmental-ocular disease networks: air pollution and meteorological drivers. A. Environmental triggers for specific ocular disorders. B. Interrelationships among ocular diseases. VRDs = Vitreoretinal Diseases; TAO = Thyroid-Associated Orbitopathy; C = Cataract; LDDs = Lacrimal Duct Diseases; U = Uveitis; G = Glaucoma; S = Strabismus; DOSA = Diseases of Ocular Surface and Appendages; ON = Optic Neuropathy; OT = Ocular Trauma; *F* = factor.

### Interrelationships among Ocular Diseases

Quantitative assessment of ophthalmic disease relationships using the number and weight of simple paths is presented in Tables 3 and 4. The distribution of these weights is illustrated in Figure S3. Analysis of the number via a simple path within clinical pathway networks revealed maximized association strength for the following directional interactions: VRD → U (*n* = 42), TAO → U (*n* = 42), C → G (*n* = 24), LDD → TAO (*n* = 49), U → VRD (*n* = 29), G → ON (*n* = 32), S → U (*n* = 47), DOSA → TAO (*n* = 57), ON → TAO (*n* = 59), and OT → VRD (*n* = 30). Concurrent evaluation of simple path weights demonstrated statistically significant correlations: VRD ↔ LDD (weight = 0.13), TAO ↔ S (0.45), C ↔ LDD (0.72), LDD ↔ U (−0.08), U ↔ LDD (−0.22), G ↔ TAO (0.37), S ↔ LDD (−0.25), DOSA ↔ LDD (−0.25), ON ↔ OT (0.93), and OT ↔ TAO (0.72) (Figure 4B).

**Table 3:** Quantitative Assessment of Ophthalmic Disease Relationships via the Number of Simple Paths^a^

	VRD	TAO	C	LDD	U	G	S	DOSA	ON	OT
**VRD**	0	34	28	18	42	28	23	7	14	27
**TAO**	18	0	18	29	42	18	32	6	15	39
**C**	16	17	0	15	15	24	18	5	20	17
**LDD**	47	49	45	0	45	32	36	11	33	41
**U**	29	10	18	15	0	18	18	7	18	23
**G**	28	33	17	24	28	0	24	10	32	23
**S**	33	33	31	15	47	15	0	6	16	47
**DOSA**	44	57	41	28	48	49	44	0	37	49
**ON**	38	59	44	21	53	29	34	1	0	50
**OT**	30	20	16	13	22	28	30	8	26	0

a VRDs = Vitreoretinal Diseases; TAO = Thyroid-Associated Orbitopathy; C = Cataract; LDDs = Lacrimal Duct Diseases; U = Uveitis; G = Glaucoma; S = Strabismus; DOSA = Diseases of Ocular Surface and Appendages; ON = Optic Neuropathy; OT = Ocular Trauma.

**Table 4:** Quantitative Assessment of Ophthalmic Disease Relationships via the Weight of Simple Paths^a^

	**TAO**	**C**	**LDD**	**U**	**G**	**S**	**DOSA**	**ON**	**OT**
**VRD**	–0.01622	–0.06465	0.125709	–0.01567	–0.07648	–0.1808	0.019512	–0.01231	0.020885
	**VRD**	**C**	**LDD**	**U**	**G**	**S**	**DOSA**	**ON**	**OT**
**TAO**	–0.28661	0.0954	–0.25295	0.001979	0.017272	0.45018	–0.02027	0.026214	–0.01287
	**VRD**	**TAO**	**LDD**	**U**	**G**	**S**	**DOSA**	**ON**	**OT**
**C**	0.010951	0.0711	0.724745	–0.07901	–0.02466	–0.10208	–0.00577	0.023698	0.069193
	**VRD**	**TAO**	**C**	**U**	**G**	**S**	**DOSA**	**ON**	**OT**
**LDD**	–0.0013	0.04129	–0.06989	–0.07684	–0.01275	–0.05892	–0.00878	–0.00045	0.039911
	**VRD**	**TAO**	**C**	**LDD**	**G**	**S**	**DOSA**	**ON**	**OT**
**U**	0.028953	–0.02443	0.103106	–0.22465	0.038421	0.219152	0.028773	–0.00464	–0.05659
	**VRD**	**TAO**	**C**	**LDD**	**U**	**S**	**DOSA**	**ON**	**OT**
**G**	–0.16561	0.368154	0.077206	–0.18315	–0.01773	0.189713	–0.08864	0.022782	0.003893
	**VRD**	**TAO**	**C**	**LDD**	**U**	**G**	**DOSA**	**ON**	**OT**
**S**	0.07105	–0.0429	0.086692	–0.24625	0.020597	–0.03548	–0.01216	0.006738	–0.0373
	**VRD**	**TAO**	**C**	**LDD**	**U**	**G**	**S**	**ON**	**OT**
**DOSA**	–0.06992	–0.01738	0.061956	–0.25131	0.220944	0.145173	0.133011	–0.19721	–0.13578
	**VRD**	**TAO**	**C**	**LDD**	**U**	**G**	**S**	**DOSA**	**OT**
**ON**	–0.32609	0.670534	0	–0.18588	–0.02725	0.382964	0.301861	–0.12722	0.925965
	**VRD**	**TAO**	**C**	**LDD**	**U**	**G**	**S**	**DOSA**	**ON**
**OT**	–0.20755	0.724146	0.087365	–0.29083	–0.01973	0.093875	0.325996	–0.06555	0.051871

a VRDs = Vitreoretinal Diseases; TAO = Thyroid-Associated Orbitopathy; C = Cataract; LDDs = Lacrimal Duct Diseases; U = Uveitis; G = Glaucoma; S = Strabismus; DOSA = Diseases of Ocular Surface and Appendages; ON = Optic Neuropathy; OT = Ocular Trauma.

## Discussion

This study demonstrates a systematic FCM application by integrating over five years of ophthalmic hospitalization data with high-resolution environmental parameters. Our approach quantitatively elucidates the intricate influence networks through which meteorological and pollutant factors impact various ocular diseases. Key findings include significant associations between environmental conditions and diseases, as well as the identification of interactive comorbidity networks.

### Environmental Risk Factors and Ocular Disease Associations

The results of our study underscore the significant regulatory roles that environmental elements exert on distinct ocular disorders.

#### Glaucoma and Climatic Influences

Prior investigations have established that environmental factors heighten the risk of acute glaucoma episodes, with fluctuations in atmospheric pressure and temperature identified as critical contributors.^28–30^ Furthermore, airborne pollutants, including PM_2.5_, nitrogen oxides (NO_*x*_), and carbon monoxide (CO), have been correlated with an increased incidence of glaucoma.^31,32^ Our findings demonstrated significant correlations between glaucoma and environmental variables, notably minimum temperature and dew point. These insights enhance our understanding of how climatic conditions may impact ocular health and emphasize the necessity for incorporating environmental considerations into glaucoma risk assessments.^28^ Notably, studies employing advanced techniques like wavelet neural networks have improved the accuracy of PM2.5 and ozone predictions, highlighting the value of integrating such models into health impact assessments.^33,34^ The association with the minimum temperature suggests that lower temperatures may contribute to the pathophysiology of glaucoma by inducing ocular vasoconstriction, potentially resulting in variations in intraocular pressure. This mechanism is consistent with existing literature documenting that cold environmental conditions can negatively influence ocular blood flow and aqueous humor dynamics.^28,35^ Previous studies have indicated that environmental humidity can exacerbate symptoms in individuals with preexisting ocular conditions, potentially increasing their risk for complications.^36^ Moreover, the observed correlation with the dew point may indicate that elevated moisture levels could disrupt corneal osmotic equilibrium, potentially impairing endothelial functionality and diminishing aqueous outflow efficiency.

#### DOSA and Climatic Sensitivity

In this research, we uncovered a significant association between environmental factors and DOSA. Our analysis revealed a strong correlation between precipitation levels and the incidence of DOSA, suggesting that increased rainfall, which typically elevates ambient humidity, has important implications for ocular health. Epidemiological data indicate that relative humidity has a negative correlation with the risk of allergic conjunctivitis. Lower humidity levels can exacerbate the dispersion of airborne particulate matter and allergens, contributing to tear film instability and an increased risk of allergic reactions.^37^ Besides, severe rainfall events can lead to flooding that may contaminate water sources. Such contamination facilitates the spread of infectious agents, potentially resulting in heightened rates of infectious conjunctivitis and keratitis.^38^ Overall, our analysis highlights the necessity of considering the impact of environmental conditions, such as precipitation, on the prevalence and types of ocular diseases, especially in the context of understanding seasonal variations.

#### Lacrimal Duct Diseases and Wind Exposure

A novel association between LDD and wind exposure was identified in our study. Previous studies have shown that the etiology of lacrimal duct diseases includes congenital abnormalities, such as a persistent Hasner membrane, acquired obstructions caused by inflammation, trauma, or tumors, and functional disorders like tear pump dysfunction.^39^ To date, few studies have explored the impact of environmental factors on the development of lacrimal duct diseases. Indeed, an elevated wind speed may accelerate tear film evaporation, thereby compromising the ocular surface barrier and potentially triggering inflammation in the lacrimal drainage system. This finding underscores the need for further exploration into environmental stressors impacting ocular health.

#### Vitreoretinal Diseases and Atmospheric Factors

This study offers new insights into the associations between vitreoretinal diseases, including diabetic retinopathy, retinal detachment, and retinal vascular occlusion, and environmental factors such as atmospheric pressure and dew point. Clinical evidence supports associations between vitreoretinal diseases and systemic/metabolic factors, including diabetes mellitus,^40^ hypertension,^41^ myopia^42^ and leukemia,^43^ among others.^44,45^ However, limited research has investigated the relationship between vitreoretinal diseases and environmental factors. Studies have found that levels of inflammatory mediators in the vitreous humor, such as IL-6, CXCL8, and CCL2, are significantly elevated in various retinal diseases, particularly in proliferative vitreoretinopathy (PVR) and inflammatory disorders.^46^ Importantly, the present study revealed a positive correlation between these climatic variables (atmospheric pressure and dew point) and the prevalence of vitreoretinal diseases, suggesting that variations in atmospheric conditions may affect the initiation and progression of these disorders by promoting local inflammatory responses or altering the blood-retinal barrier function. This finding represents a significant advancement in understanding the multifactorial nature of vitreoretinal diseases as it highlights how environmental factors interact with biological mechanisms to affect ocular health.

#### Optic Neuropathy and Environmental Triggers

ON encompasses a spectrum of conditions affecting the optic nerve, including those caused by ischemia, inflammation, elevated intracranial pressure, compression and infiltration, toxins, nutritional deficiencies, and hereditary diseases.^47^ Limited direct investigations have examined the correlation between optic neuropathy and environmental variables. This study presents groundbreaking findings regarding the relationship between ON and environmental factors, particularly air pollution and meteorological conditions. A particularly important limitation is the potential for unmeasured confounding. The FCM framework does not explicitly adjust for key individual-level risk factors that could influence both environmental exposures and the ophthalmic outcome. Future studies should strive to incorporate these critical variables through primary data collection or linkage with detailed socioeconomic and medical databases to improve causal inference with PM_2.5_, SO_2_, CO, NO_2_, and O_3_ warrant careful mechanistic interpretation. These opposing effects may be attributed to several biological and physicochemical factors. First, the distinct particle sizes and chemical compositions of these pollutants likely lead to different deposition patterns and biological interactions in the ocular tissues.^32,48^ While larger PM10 particles primarily induce mechanical irritation and inflammatory responses in anterior ocular structures, finer PM2.5 particles and gaseous pollutants may penetrate deeper and trigger different pathological pathways through oxidative stress or vascular dysfunction mechanisms.^49,50^ Second, the complex atmospheric interactions and potential confounding among pollutants might create apparent opposing associations—for instance, certain pollutants could serve as proxies for unmeasured environmental cofactors.^51^ Third, the negative associations observed for some pollutants might indicate the presence of competitive binding mechanisms or threshold effects in neuro-inflammatory pathway.^52^ From a methodological perspective, these divergent associations could also be influenced by exposure misclassification patterns that vary by pollutant type,^53^ or by the FCM framework’s sensitivity to nonlinear relationships.^54^ While our current analysis cannot definitively distinguish between these possibilities, these findings highlight the complex nature of pollutant-ocular interactions and underscore the need for further investigation using more refined exposure assessments and mechanistic studies. Furthermore, our analysis revealed that environmental conditions such as the dew point, precipitation, maximum temperature, and visibility were negatively correlated with ON. This innovative approach not only enhances our understanding of the environmental determinants of ON but also emphasizes the need for multidisciplinary studies that integrate ophthalmology and environmental science.

#### Cataract Formation and Environmental Conditions

Numerous studies have identified long-term smoking^55^ and exposure to biomass fuel (BMF) smoke^32,56^ as primary risk factors for cataract formation. Our study demonstrated significant environmental correlations with cataract development: positive associations with CO, visibility, and temperature, alongside negative associations with the dew point (a critical indicator of atmospheric moisture) and precipitation. While prior research has established links between CO and other ocular conditions such as dry eye disease,^57^ conjunctivitis^58^ and retinal disorders,^59^ its specific role in cataractogenesis has remained underexplored. Our results underscore CO as a novel environmental risk factor for cataract formation. Specifically, elevated ambient temperature is now understood to accelerate lens protein denaturation through increased oxidative stress and altered aqueous humor dynamics.^60^ Notably, lower humidity levels and higher temperatures have been associated with a greater risk of developing cataracts.^61^ A recent study on the evolution and driving mechanisms of eco-environmental quality across different urbanization stages further illustrates how anthropogenic environmental changes drive disease patterns,^62^ aligning with our findings on cataract risks. Our findings revealed that dew point levels and precipitation were negatively correlated with the incidence of cataracts, suggesting that dry environmental conditions may contribute to the pathophysiological processes leading to cataract development.

### Clinical Significance of Disease Interaction Networks

The eye functions as a relatively autonomous visual organ comprising various ocular tissues that are anatomically adjacent and functionally interconnected. Clinical findings and research have suggested that dry eye frequently coexists with conditions such as meibomian gland dysfunction, allergic conjunctivitis, and other corneal disorders.^63,64^ Besides, individuals with high myopia are at an elevated risk for developing cataracts, glaucoma, and vitreoretinal diseases.^65^ Moreover, the development of a cloudy and swollen lens is widely thought to precipitate acute glaucoma.^66,67^ The findings presented above suggest potential interrelationships among various ocular diseases. While the associations between some common ocular diseases (such as the connection between cataracts and glaucoma) are well-established, numerous other possible associations remain poorly understood.

In the present study, FCMs were utilized to clarify the interrelationships among ten principal ocular diseases, uncovering significant patterns that enhance our comprehension of their clinical pathways. Notably, an analysis of connection strengths within clinical pathway networks revealed maximal association strengths for several directional interactions. For example, robust associations were identified between VRD and uveitis and between thyroid-associated orbitopathy and uveitis (both exhibiting an interaction weight of 42). This finding suggests potential interconnectedness, wherein the presence of one condition may elevate the risk of the other, possibly indicating shared underlying mechanisms. Besides, cataracts demonstrated a significant interaction with glaucoma, suggesting that patients diagnosed with cataracts may possess an increased likelihood of developing glaucoma, as reflected by a path count of 24. This relationship emphasizes the urgent necessity of comprehensive management strategies for patients with cataracts to facilitate monitoring for concurrent glaucoma.

The concurrent evaluation of weights via a simple path further corroborated these findings, revealing significant correlations that provide insights into the nature of these relationships. Notably, TAO exhibited a significant correlation with Strabismus (weight = 0.45), Glaucoma (weight = 0.37), and OT (weight = 0.72). These results consistently aligned with documented clinical observations, which indicate that severe TAO can lead to complications such as ocular misalignment and increased intraocular pressure with potential progression to optic nerve damage. Furthermore, TAO displayed substantial network connectivity, as demonstrated by its multiple direct associations with other ocular diseases (ON → TAO: *n* = 59; LDD → TAO: *n* = 49; DOSA → TAO: *n* = 57). Consequently, the analysis identifies TAO as a potential central hub supported by multiple quantitative metrics that highlight its pivotal role in the disease interaction network. The strikingly high correlation between ON and OT (weight = 0.93) emphasizes the impact of traumatic events on optic nerve health, emphasizing the need for vigilant monitoring and management of patients with a history of ocular trauma. Overall, our findings elucidate critical pathways among ocular diseases, suggesting that multidisciplinary approaches to treatment and management may improve patient outcomes by addressing these interconnected conditions.

Our findings reveal both consistencies and enlightening divergences when compared with traditional epidemiological methods such as linear regression, case-control studies, and time-series analyses. In terms of consistencies, our FCM network analysis confirmed established environment-disease associations: the risk effects of temperature and dew point on glaucoma;^28,36^ the positive correlation between ambient temperature and cataract incidence;^61^ and the significant association between ocular surface diseases (DOSA) and precipitation levels.^37,38^ However, the most notable differences emerged in the complex association patterns between optic neuropathy (ON) and pollutants: unlike the linear relationships typically reported in traditional studies, we found PM10 to be positively correlated with ON, whereas PM2.5, SO2, CO, NO2, and O3 showed negative correlations, revealing the complexity of pollutant-ocular interactions. More importantly, compared to traditional linear models, our FCM framework successfully captured bidirectional relationships and network effects among diseases, identifying thyroid-associated orbitopathy (TAO) as a central hub in the disease network—a critical feature that conventional methods failed to detect. These findings not only demonstrate the innovative value of our methodology but also elucidate the complex nature of environmental etiology in ocular diseases, providing new directions for subsequent research.

#### Limitations

This study has several limitations that should be considered in interpreting the results. As a retrospective analysis using real-world clinical and environmental data, it identifies associations but cannot establish causal relationships between environmental exposure and ophthalmic diseases. The findings are primarily hypothesis-generating, serving to guide future investigations rather than confirm causality.

A particularly important limitation is the potential for unmeasured confounding. The FCM framework does not explicitly adjust for key individual-level risk factors that influence both environmental exposures and ophthalmic outcomes. These include socioeconomic status, occupational exposures, smoking history, genetic susceptibility, and comorbidities such as diabetes and hypertension. Furthermore, the model does not account for temporal structures in environmental exposures, including long-term trends, seasonal patterns, and seasonal variations, which may introduce additional bias into the observed associations. The absence of these variables in our analysis means that residual confounding may exist, potentially biasing the observed associations. For example, if individuals with lower socioeconomic status are more likely to experience higher pollutant exposure and poorer ocular health outcomes, this could create a spurious association between environmental factors and eye diseases. While the complex, multidirectional nature of FCMs provides some capability to account for interrelated factors, the lack of explicit adjustment for these specific confounders remains a limitation. Future studies should strive to incorporate these critical variables through primary data collection or linkage with detailed socioeconomic and medical databases and should consider implementing time-series analytical approaches^68^ to better account for temporal variations to improve causal inference.

Additionally, the analysis involved numerous variables and pathways, which inherently raises the issue of multiple testing. Given the exploratory nature of this study aimed at hypothesis generation, we did not apply statistical corrections (e.g., Bonferroni or FDR) to *p*-values or effect estimates. This uncorrected approach may increase the risk of false positives (Type I errors). Therefore, the reported associations should be interpreted with caution as indicative rather than definitive, and they require validation in independent cohorts or through experimental studies to confirm their reliability and generalizability. The absence of such corrections means that some findings may be due to chance, and the results should be viewed as generating leads for future research rather than confirming etiological pathways.

Exposure assessment represents a significant limitation in this study. Our reliance on regional monitoring station data may lead to exposure misclassification, as this area-level approach fails to adequately capture individual-level variability arising from factors such as time-activity patterns, indoor–outdoor differences, and occupational settings. The spatial heterogeneity of pollutants and meteorological factors means that regional averages are a poor proxy for true personal exposure levels. Furthermore, the use of daily averages matched to hospital admission dates cannot fully account for microenvironmental variations and exposure duration. Although advanced spatiotemporal modeling techniques could refine these estimates, they were beyond the scope of this retrospective analysis due to data and resource constraints. Such nondifferential misclassification generally attenuates effect estimates toward the null, potentially resulting in an underestimation of the true association strengths. Therefore, future studies should prioritize the integration of personal exposure monitors with spatiotemporal modeling techniques to minimize this bias.

Additional constraints include the emphasis on inpatient data, omitting outpatient cases with milder manifestations, and potentially underestimating environmental effects on early disease stages. This reliance on inpatient data introduces potential selection bias, as it may not represent the full spectrum of ophthalmic disease severity or subtypes, limiting the generalizability of our findings to broader populations, including outpatient or community-based settings. Furthermore, the analysis included disease categories with varying sample sizes. The marked imbalance in case numbers, particularly for diseases with lower prevalence, may introduce estimation uncertainty in integrated Model 11, which simultaneously evaluates all diseases. Although the fuzzy cognitive mapping framework is relatively robust to such variability, findings for diseases with smaller sample sizes should be interpreted with greater caution, primarily as hypothesis-generating. Future validation of these preliminary associations and improved model stability across all disease categories will require larger, multicenter study cohorts. Finally, the interpretation of the FCM weights necessitates further validation through supplementary studies focusing on molecular mechanisms. Future research should leverage advances in climate prediction and air quality modeling (e.g., deep learning and LSTM techniques^69,70^ ) to refine exposure assessments and establish causal thresholds.

## Conclusions

The present study established a novel dynamic network using FCM to model interactions between ophthalmic diseases and environmental parameters. The approach revealed potential influences of temperature, dew point, and pollutants on specific ocular pathologies and uncovered systemic interdisease relationships. These findings, which are exploratory and indicative, provide a preliminary foundation for hypothesizing precision prevention strategies targeting environmentally aggravated ocular conditions. By proposing a shift from isolated disease management to an integrated approach that encompasses environment-disease network interventions, this work advances the field while underscoring the need for future validation to establish causal inference and clinical applicability.

## Supplementary Material



## Data Availability

The code for training and testing the models in the current research, as well as the datasets generated or analyzed during this study, is available from the corresponding author upon reasonable request.

## Notes

Tables (S1–S6) and Figures (S1–S3), including statistical results of environmental factors and ocular diseases; algorithm parameters in differential evolution and models 1–11; MAE of all models; population age and sex information; Variance Inflation Factor (VIF) of the data set; distribution of simple path weights; and disease interaction network evaluation (with full terminology definitions for all ocular disease abbreviations) (PDF)
