# Supplementary material for: Environmental Triggers and Ocular Disease Networks: Analyzing the Impact of Air Pollutants and Meteorological Factors Using Fuzzy Cognitive Maps
Source: Environ Health (Wash). 2026 Jan 14;4(5):1012–25. doi: 10.1021/envhealth.5c00522 (PMC13185060; doi:10.1021/envhealth.5c00522)
Supplement: Supplementary file 1 [file eh5c00522_si_001.pdf]

Environmental Triggers and Ocular Disease Networks: Analyzing the Impact of Air Pollutants and Meteorological Factors Using Fuzzy Cognitive Maps

Li Zhang[1,2,3†], Fabao Xu[4†], Haixiang Jiang[5†], Yaxin miao[1], Yi Xiang[1], Lu Zhang[1], Qing Huang[1], Deying Yu[6], Meijia Wang[7], Xu Wang[8], Shiqiang Li[4], Boxuan Song[4], Zhiwen Li[4], Xueying Yang[4], Jing Wei[9], Jianqiao Li[4*], Chengcheng Zhang[10*], Kai Zhang[11*], Yong Wang[2,3*]

1 Department of Ophthalmology, The Central Hospital of Wuhan, Tongji Medical College, Huazhong University of Science and Technology, 26 Shengli Street, Jiang'an District, Wuhan, Hubei Province 430014, China.

2 Aier Eye Hospital of Wuhan University (Wuhan Aier Eye Hospital), No. 481, Zhongshan Road, Wuchang District, Wuhan, Hubei Province, 430060, China.

3 Wuhan Aier Eye Institute, No. 790, Minzhu Road, Wuchang District, Wuhan, Hubei Province 430060, China.

4 Department of Ophthalmology, Qilu Hospital, Shandong University, 107 West Wenhua Road, Lixia District, Jinan, Shandong Province 250011, China.

5 Haixiang Eye Hospital, 44 Changle West Road, Xincheng District, Xi'an, Shaanxi Province 710032, China.

6 Department of Ophthalmology, University Malaya Medical Centre, Jalan Profesor Diraja Ungku Aziz, Lembah Pantai, Kuala Lumpur, 59100, Malaysia.

7 School of Electronic Information and Artificial Intelligence, Shannxi University of Science & Technology, Xi'an Weiyang University Park, 1 Weiyang Road, Weiyang District, Xi'an, Shaanxi Province 710021, China.

8 School of Software, Shanxi Agricultural University, 1 Mingxian South Road, Taigu District, Jinzhong, Shanxi Province 030801, China.

9 MEEKL-AERM, College of Environmental Sciences and Engineering, Institute of Tibetan Plateau, and Center for Environment and Health, Peking University, 5 Yiheyuan Road, Haidian District, Beijing 100871, China.

10 Medical Genetics Center, Maternal and Child Health Hospital of Hubei Province, 745 Wuluo Road, Hongshan District, Wuhan, Hubei Province 430070, China.

11 Gyenno Science Co. Ltd., 18F, Tower B, Galaxy World, 1 Changfa Road, Nanshan District, Shenzhen, Guangdong Province 518055, China.

†These authors contributed equally to this work.

*Co-corresponding authors:

Yong Wang, 481 Zhongshan Road, Wuchang District, Wuhan City, Hubei Province 430060, China. E-mail: wangyongeye@163.com.

Jianqiao Li, No. 107 Wenhua West Road, Lixia District, Jinan City, Shandong Province 250011, China. Email: 18560087118@163.com.

Chengcheng Zhang, No. 745 Wu Luo Road, Hongshan District, Wuhan City, Hubei Province 430070, China. Email: 3842096480@qq.com.

Kai Zhang, 8th Floor, Building B2 (West Side), Creative City, Nanshan District, Shenzhen 518055, China. E-mail: Hugo88315@163.com.

Table S1. The statistical results of environmental factors and ocular diseases

	Parameter	Mean value	Standard deviation	Maximum value	Minimum value
Air pollutants	$PM_{2.5}$ (μg/m³)	38.3365	26.5732	206	0
	PM_{10} (μg/m³)	60.3004	35.0651	410	0
	SO_2 (μg/m³)	14.3553	20.1997	109	0
	CO (mg/m³)	16.7522	18.800	99	0
	NO_2 (μg/m³)	15.2931	18.4802	98	0.4000
	O_3 (μg/m³)	99.6473	47.2967	258	0
Meteorological factors	Temperature (°C)	17.7446	9.3341	34.7250	-2.9875
	Atmospheric pressure (mmHg)	759.6615	7.5687	779.9500	670.3500
	Humidity (%)	76.1419	11.1800	99.2500	29.8750
	Wind speed (m/s)	1.5895	0.8881	6.2500	0
	Minimum temperature (°C)	11.1138	9.6613	31.6125	-7.3750
	Maximum temperature (°C)	17.1276	10.9357	39.6625	-1.3000
	Visibility (km)	10.9406	6.5545	30	0.4625
	Dew point (°C)	12.8458	9.2560	27.2000	-16.1625
	Precipitation (mm)	1.6709	5.3634	59.7500	0
Ocular diseases	VRD	3.8905	3.7217	22	0
	TAO	0.0286	0.1627	1	0
	C	8.0656	8.4187	49	0
	LDD	0.1791	0.5341	5	0
	U	0.1223	0.3621	4	0
	G	0.4544	0.7337	5	0
	S	0.0459	0.2295	3	0
	DOSA	1.2644	1.4667	10	0
	ON	0.0266	0.1611	1	0
	OT	0.2447	0.5344	4	0

VRD = Vitreoretinal Diseases; TAO = Thyroid-Associated Orbitopathy; C = Cataract; LDD = Lacrimal Duct Diseases; U = Uveitis; G = Glaucoma; S = Strabismus; DOSA = Diseases of Ocular Surface and Appendages; ON = Optic Neuropathy; OT = Ocular Trauma.

Table S2. Algorithm parameter in Differential Evolution.

Item	Value
**Iterations**	1000
**Cross rate (CR)**	0.2
p	0.8
**Population size**	100

Table S3. All the algorithm parameters in Model 1-11.

		Modeling results		
**Order**	**Model**	**Mean value ± S.D.**	**Maximum value**	**Minimum value**
1st	1	0.15797±0.0089745	0.17257	0.14189
	2	0.15592±0.018829	0.19219	0.12272
	3	0.15242±0.023299	0.18295	0.094025
	4	0.16116±0.012893	0.18745	0.14157
	5	0.15154±0.020568	0.16612	0.10131
	6	0.15388±0.017374	0.16877	0.11165
	7	0.16696±0.024071	0.21691	0.13546
	8	0.14371±0.019399	0.16538	0.11404
	9	0.1465±0.035674	0.2234	0.1043
	10	0.14668±0.026471	0.17373	0.086288
	11	0.15968±0.019098	0.20086	0.14499
2nd	1	0.15845±0.023686	0.18984	0.11817
	2	0.1451±0.025352	0.19795	0.10338
	3	0.15841±0.022631	0.18718	0.11697
	4	0.15353±0.013458	0.17103	0.1381
	5	0.15465±0.019187	0.17967	0.12739
	6	0.14543±0.032583	0.18455	0.078061
	7	0.14733±0.023037	0.17618	0.094486
	8	0.14729±0.021855	0.17468	0.098917
	9	0.14574±0.02703	0.18765	0.1156
	10	0.13484±0.022258	0.16616	0.090067
	11	0.16367±0.02841	0.20464	0.12518
3rd	1	0.15797±0.0089745	0.17257	0.14189
	2	0.15592±0.018829	0.19219	0.12272
	3	0.15242±0.023299	0.18295	0.094025
	4	0.16116±0.012893	0.18745	0.14157
	5	0.15154±0.020568	0.16612	0.10131
	6	0.15388±0.017374	0.16877	0.11165
	7	0.16696±0.024071	0.21691	0.13546
	8	0.14371±0.019399	0.16538	0.11404
	9	0.1465±0.035674	0.2234	0.1043
	10	0.14668±0.026471	0.17373	0.086288
	11	0.15968±0.019098	0.20086	0.14499

Model 1-11 and Corresponding Ocular Diseases: Model 1 for Vitreoretinal Diseases (VRD) ; Model 2 for Thyroid-Associated Orbitopathy (TAO); Model 3 for Cataract (C); Model 4 for Lacrimal Duct Diseases (LDD); Model 5 for Uveitis (U); Model 6 for Glaucoma (G); Model 7 for Strabismus (S); Model 8 for Disease of Ocular Surface and Appendages (DOSA); Model 9 for Optic Neuropathy (ON); Model 10 for Ocular Trauma (OT)； Model 11 for all kinds of ocular diseases.

Table S4. MAE (Mean Absolute Error) of all models.

Model	MAE
1	0.6331
2	0.6207
3	0.4034
4	0.4888
5	0.3304
6	0.4715
7	0.6020
8	0.4628
9	0.4471
10	0.3541
11	0.6340

Table S5. The information on the age and sex of population.

Demographic		Ocular diseases										
		VRD	TAO	C	LDD	U	G	S	DOSA	ON	OT	Total cases
Sex	Male	4074	24	6827	72	136	367	44	1211	37	344	13136
	Female	3762	34	9524	29	112	554	49	1350	17	152	15845
Average age		61.59	58.12	70.19	62.62	52.29	62.97	29.91	59.01	61.93	50.27	65.89

VRD = Vitreoretinal Diseases; TAO = Thyroid-Associated Orbitopathy; C = Cataract; LDD = Lacrimal Duct Diseases; U = Uveitis; G = Glaucoma; S = Strabismus; DOSA = Diseases of Ocular Surface and Appendages; ON = Optic Neuropathy; OT = Ocular Trauma.

Table S6. The Variance Inflation Factor (VIF) of dataset.

No.	Indicator	Variance Inflation Factor (VIF)
1	PM2.5	5.40384800000000
2	PM10	5.54435900000000
3	SO2	2.93972300000000
4	CO	2.58854900000000
5	NO2	2.55002600000000
6	O3	2.81065500000000
**7**	**Temperature**	**271.397113000000**
8	Atmospheric pressure	4.97896800000000
**9**	**Humidity**	**23.8916510000000**
10	Wind speed	1.63056400000000
**11**	**Minimum temperature**	**11.7922790000000**
**12**	**Maximum temperature**	**7.26255500000000**
13	Visibility	3.78537100000000
**14**	**Dew point**	**265.670473000000**
15	Precipitation	1.30213500000000
16	VRD	1.85446100000000
17	TAO	1.01412900000000
18	C	2.07814600000000
19	LDD	1.16158100000000
20	U	1.04432300000000
21	G	1.09363700000000
22	S	1.02234600000000
23	DOSA	1.47667200000000
24	ON	1.02935300000000
25	OT	1.08300200000000

VRD = Vitreoretinal Diseases; TAO = Thyroid-Associated Orbitopathy; C = Cataract; LDD = Lacrimal Duct Diseases; U = Uveitis; G = Glaucoma; S = Strabismus; DOSA = Diseases of Ocular Surface and Appendages; ON = Optic Neuropathy; OT = Ocular Trauma.

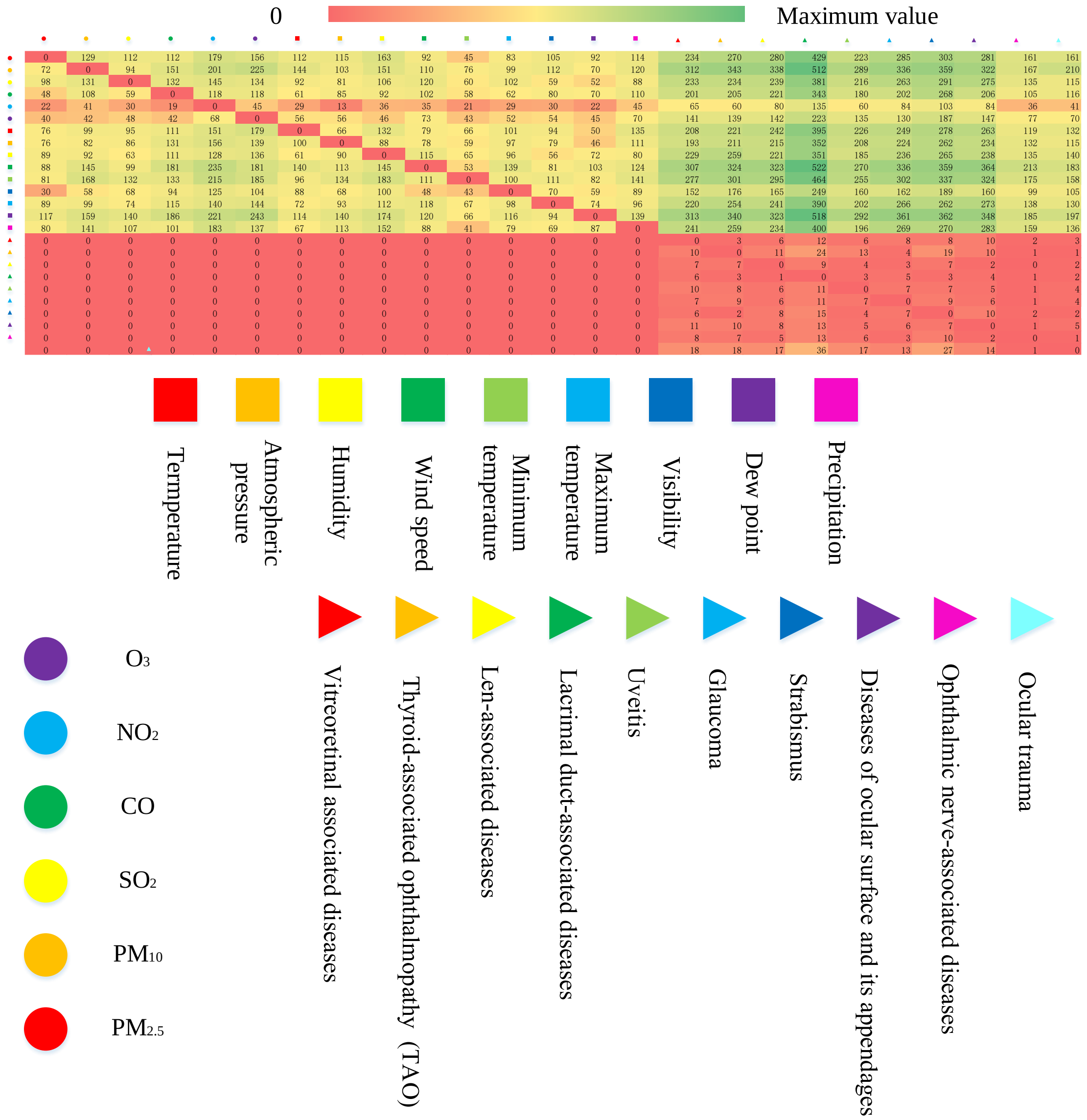

Figure S1. All the number of simple paths in Model 11.

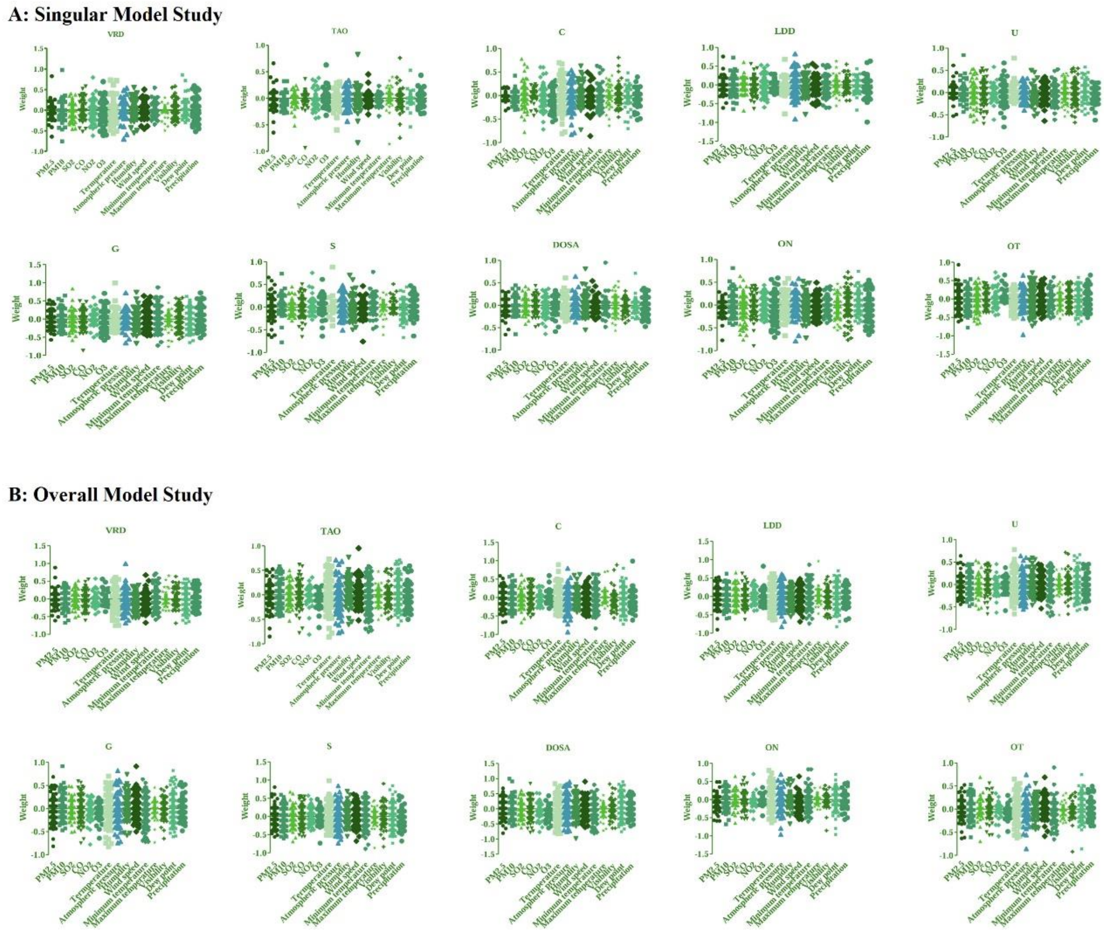

Figure S2. Distribution of simple path weights in individual models (A) and the comprehensive model (B) for evaluating environmental factor impacts on ocular diseases. VRD = Vitreoretinal Diseases; TAO = Thyroid-Associated Orbitopathy; C = Cataract; LDD = Lacrimal Duct Diseases; U = Uveitis; G = Glaucoma; S = Strabismus; DOSA = Diseases of Ocular Surface and Appendages; ON = Optic Neuropathy; OT = Ocular Trauma；

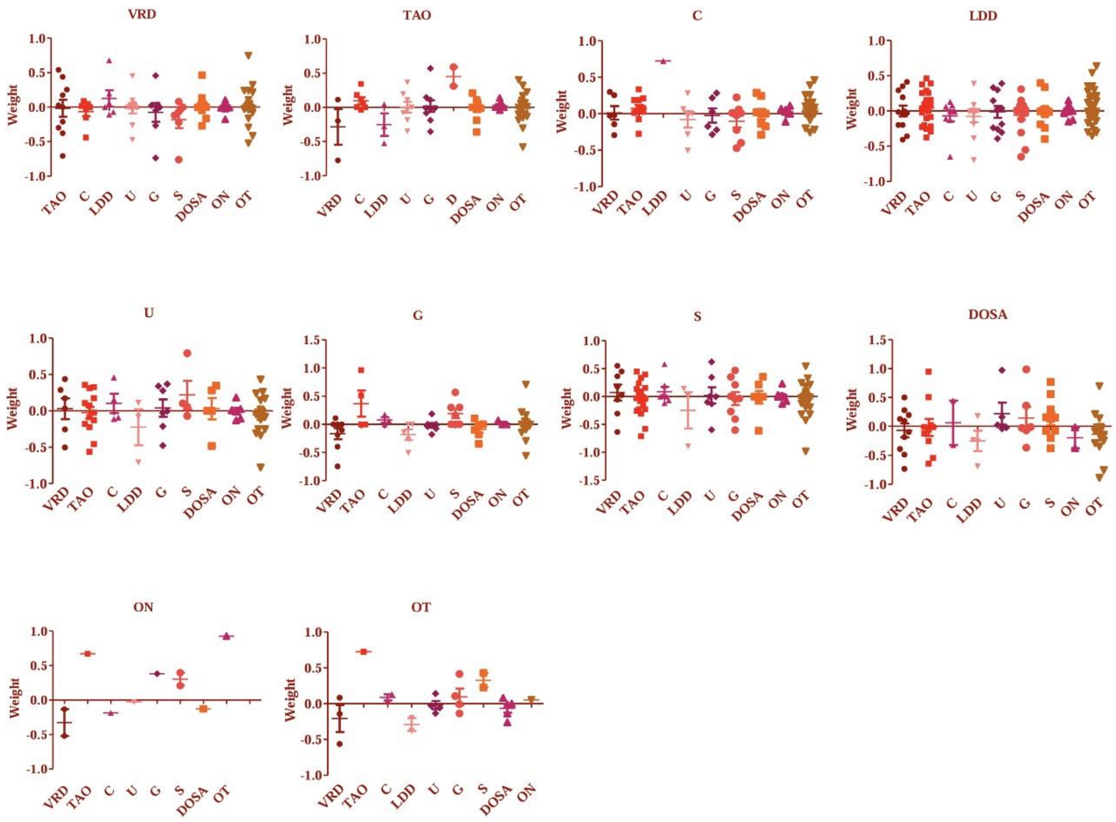

Figure S3. Distribution of simple path weights in Model 11 for evaluating disease interaction networks. VRD = Vitreoretinal Diseases; TAO = Thyroid-Associated Orbitopathy; C = Cataract; LDD = Lacrimal Duct Diseases; U = Uveitis; G = Glaucoma; S = Strabismus; DOSA = Diseases of Ocular Surface and Appendages; ON = Optic Neuropathy; OT = Ocular Trauma；
